# Rapid Separation of Enantiomeric Impurities in Chiral Molecules by a Self-Referential Weak Measurement System

**DOI:** 10.3390/s18113788

**Published:** 2018-11-06

**Authors:** Yang Xu, Lixuan Shi, Tian Guan, Suyi Zhong, Xuesi Zhou, Dongmei Li, Cuixia Guo, Yuxuan Yang, Xiangnan Wang, Zhangyan Li, Yonghong He, Luyuan Xie, Zonghan Gan

**Affiliations:** 1 Institute of Optical Imaging and Sensing, Shenzhen Key Laboratory for Minimal Invasive Medical Technologies, Graduate School at Shenzhen, Tsinghua University, Shenzhen 518055, China; xuyang17@mails.tsinghua.edu.cn (Y.X.); slx16@mails.tsinghua.edu.cn (L.S.); zhongsy18@mails.tsinghua.edu.cn (S.Z.); zhouxs17@mails.tsinghua.edu.cn (X.Z.); Guocx14@mails.tsinghua.edu.cn (C.G.); yx-yang16@mails.tsinghua.edu.cn (Y.Y.); wxn16@mails.tsinghua.edu.cn (X.W.); li-zy17@mails.tsinghua.edu.cn (Z.L.); xly16@mails.tsinghua.edu.cn (L.X.); 2 Department of Physics, Tsinghua University, Beijing 100084, China; 3 School of Medicine, Tsinghua University, Beijing 100084, China; 4 Center for Optics & Optoelectronics Research, Collaborative Innovation Center for Information Technology in Biological and Medical Physics, College of Science, Zhejiang University of Technology, Hangzhou 310023, China; ldm20010@163.com; 5 Division of biomedical engineering, University of Glasgow, 89 Dumbarton Rd, Glasgow G12 8QQ, UK; 2351980g@student.gla.ac.uk

**Keywords:** weak measurement, self-reference, common light path system, enantiomeric impurity content detection

## Abstract

We propose a self-referential fast detection scheme for a frequency domain weak measurement system for the detection of enantiomeric impurities in chiral molecules. In a transmissive weak measurement system, the optical rotation (OR) is used to modify the pre-selected polarization state and the post-selection polarization state. We obtained the sum and difference of the optical rotations produced by the sample and the standard by rotating the quarter wave plate in the system. Then, we estimate the ratio of chiral molecules to enantiomeric impurities using the ratio of the central wavelength shifts caused by the addition and subtraction states described above. In this paper, our system has an optical resolution of 1.88 × 10^−5^°. At the same time, we completed the detection of the ratio of the two substances in the mixture of L-proline and D-proline in different proportions, which proved that our system can quickly detect the content of enantiomeric impurities in chiral molecules.

## 1. Introduction

Chirality is a universal phenomenon in nature. There is a large number of chiral molecules in nature and beings. The study of chiral molecules plays an important role in life, pharmaceutical, and material science [1]. As an important foundation of life activities, biological macromolecules and many active substances acting on receptors have chiral characteristics, such as enzymes, carriers, receptors, plasma proteins, and polysaccharides. The enantiomers of chiral molecules vary widely in biological, physiological, and pharmacological activities, and even the opposite. Therefore, it is of great significance to obtain a single enantiomer for physiological and pharmacological studies [2]. At present, enantiomer impurities are often contained in chiral molecules, especially in fully synthesized chiral molecules. Therefore, the detection of isomer impurity in chiral substances has become a widespread concern.

Because the main mark of a chiral molecule is optical rotation (OR) [3,4], chiral molecules can be recognized by their optical rotation. Optical detection technology is popular due to its advantages of being nondestructive, real-time, low cost, and having simple operation. But, traditional OR detection technology has the shortcoming of low precision, i.e., its precision is only 0.001 degrees [5]. Therefore, while preserving the advantages of OR detection, improving the optical detection accuracy based on OR has become an urgent problem.

The method of quantum weak measurement was first proposed by Aharonov, Albert, and Vaidman in 1988 [6] and implemented by Ritchie et al. in 1991 [7]. By adjusting the pre-selected state and the post-selected state and maintaining a small interaction intensity, the method can magnify the feature values many times. The process that magnifies the parameters we actually need is called weak value amplification (WVA). This theory not only provides a deeper explanation of quantum physics, but also presents the potential of precise measurement. It has been widely used in some high-precision physical measurements, such as observation of the rotating Hall effect of light [8], beam deflection measurement [9,10], ultrasensitive phase estimation [11], velocity measurements [12], and distinguishing between two predetermined close vectors [13], the relative advantages of implementing weak-value-based metrology versus standard methods [14]. In 2010, Brunner et al. studied the effect of weak values’ real part and imaginary part on their amplification by using the longitudinal phase difference of weak measurement. It was proved that the detection accuracy of weak measurement in frequency domain can be 3 orders of magnitude higher than that of traditional interferometry and weak measurement in the time domain [15]. Therefore, the frequency domain weak measurement method has gradually become the focus. The frequency-domain weak measurement method has also been applied to a variety of biological chemistry sensors, such as the combination of weak measurements and Mach–Zehnder interferometers to measure blood glucose concentrations in mice [16], and combination of weak measurement techniques and total internal reflection (TIR) sensors to realize interaction with biomacromolecules [17] and a molecularly-imprinted polymer (MIP) sensor based on weak measurement technology [18]. These applications not only realize the real time application of molecular concentration change or mutual reaction, but also greatly improve the sensitivity of these sensors [19]. According to the weak measurement theory, the excursion of the output probe can be caused not only by the interaction between the system and equipment, but also by the preselection angle. Therefore, the weak measurement theory can be used to improve the accuracy of OR detection [20]. In our previous work, we also completed the detection of chiral molecules and their enantiomers using weak measurement systems, and the determination of proline and enantiomers in different environments. We also completed the detection of molecular chirality and its concentration using a weak measurement system [21,22]. These methods are capable of rapidly determining the left and right chirality of chiral molecules and their concentrations, but it is relatively cumbersome to determine the ratio of chiral molecules to their enantiomers in a sample. Therefore, a sensor system capable of rapidly determining the ratio of enantiomeric impurities in chiral molecular samples needs to be proposed.

In this paper, we present a self-referential weak measurement system for the rapid detection of enantiomeric impurities in chiral molecules. We modify the angle of the selected state before and after the weak measurement system by OR, and then adjust the quarter wave plate (QWP) angle of the system to obtain the sum and difference of the change of the angle of the solution in the two sample cells in the system to the selected state before and after the system. Thus, the ratio of chiral molecules to their enantiomers in the sample is calculated. In our work, taking a pair of valine enantiomers as an example, we placed a standard sample in the back sample cell: 1 g/L of D-valine, and placed the sample to be tested in the former sample cell: 1 g/L of L- and D-valine solution. By rotating the QWP, the sum and difference of the optical rotation changes caused by the test article and the standard product are obtained, and the ratio of L-valine and D-valine in the sample is quickly obtained by the ratio of the sum to the difference. In this paper, the detection of a system’s optical rotation can reach 1.88 × 10^−50^. The experiment proves that this self-based weak measurement sensor has the advantages of being fast, simple, low cost, and having high stability and robustness, and is of great significance in the detection of drugs and foods.

## 2. Theory

In our optical system, we measure the photon circular polarization operator A with eigenvalues 1 and −1 for the two orthogonal circular polarizations. The Hamiltonian of our system can be expressed as:

(1) H=g(t)PA

of which *g*(*t*) indicates the strength of coupling satisfying ∫g(t)dt=k, and *P* is the photon momentum. We can obtain a measurement result with higher precision by using the imaginary part of the weak value of *A*. The transversal shift is created by the phase difference of two circular polarized lights we introduce with optical instruments in the light path. In a frequency-domain weak measurement system, the interacting Hamitonian is determined by the phase difference, and pre- and post-selection states. Within a certain adjustable range, total phase difference between circular polarized lights can be used to generate the interaction of weak measurement, corresponding with certain pre- and post-selection states.

**Figure 1:**
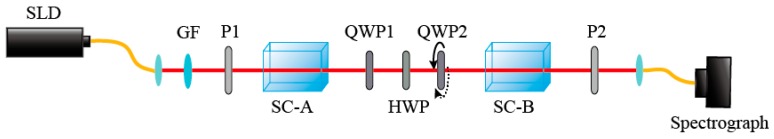
The schematic diagram of the weak measurement system.

As shown in Figure 1, our optical path system consists of a super-luminescent diode (SLD), lens, Gaussian filter (center wavelength 830 nm, FWHM 40 nm, Thorlabs Inc., Shanghai, China), pre-selected polarizer, sample cell A (SC-A), quarter wave plate (QWP), half wave plate, quarter wave plate, sample cell B (SC-B), post-selected polarizer, lens, and spectrometer (OCEAN VIEW, HR4000, Shanghai, China). Among them, the angle of the front selected polarizer is horizontal and the latter one is vertical. The angle of the quarter wave plate and half wave plate is close to the horizontal direction.

In order to study the small phase difference between L- and R-circular polarized light caused by different optical medium, we transform the linear polarization eigenstates into circular polarized eigenstates by the following relations [23,24]

(2) $$|H\rangle =\frac{\sqrt{2}}{2}\left(|+\rangle +|-\rangle \right),|V\rangle =\frac{\sqrt{2}i}{2}\left(|-\rangle -|+\rangle \right)$$

where |H〉, |V〉, |+〉, and |−〉 represent the eigenstates of horizontal linear polarized light, verticallinear polarized light, right circular polarized light, and left circular polarized light, respectively. The pre- and post-selecting polarization states can be represented by circular polarized light as

(3) $$|\psi_{pre}\rangle =|H\rangle =\frac{\sqrt{2}}{2}|-\rangle +\frac{\sqrt{2}}{2}|+\rangle$$

(4) $$|\varphi_{post}\rangle =|V\rangle =\frac{\sqrt{2}i}{2}|-\rangle -\frac{\sqrt{2}i}{2}|+\rangle$$

After the pre-polarizer, the light passes through SC-A, a set of wave plates and SC-B.

The state of light before and after the half wave plate can be now represented as

(5) $$|\psi \rangle =\frac{\sqrt{2}}{2}e^{-i\alpha }\hat{Q}|-\rangle +\frac{\sqrt{2}}{2}e^{i\alpha }\hat{Q}|+\rangle$$

(6) $$|\varphi \rangle =\frac{\sqrt{2}i}{2}e^{i\beta }|-\rangle -\frac{\sqrt{2}i}{2}e^{-i\beta }|+\rangle$$

where the optical rotation of SC-A is *α*, the optical rotation of SC-B is *β*, and $\hat{Q}$ represents the operator of the wave plates. Since the wave plates causes the phase difference of linear polarized light, we can use the modulating wave plate method to change the effect of SC-A and SC-B on the measurement results. We first adjust the slide set so that the phase difference between *H* and *V* polarized light become as △=2π+2θ,θ≪1, and the operator $\hat{Q}$ of wave plate group can be expressed as

(7) $$\hat{Q}=e^{-i\theta }|H\rangle \langle H|+e^{i\theta }|V\rangle \langle V|=\cos\theta |-\rangle \langle -|+\cos\theta |+\rangle \langle +|+i\sin\theta |+\rangle \langle -|+i\sin\theta |-\rangle \langle +|$$

The two-state vector 〈ψ||φ〉 of circular polarization is

(8) $$|\psi \rangle =\frac{\sqrt{2}}{2}\left(\cos\theta e^{-i\alpha }+i\sin\theta e^{i\alpha }\right)|-\rangle +\frac{\sqrt{2}}{2}\left(\cos\theta e^{i\alpha }+i\sin\theta e^{-i\alpha }\right)|+\rangle$$

(9) $$\langle \varphi |=-\frac{\sqrt{2}i}{2}e^{-i\beta }\langle -|+\frac{\sqrt{2}i}{2}e^{i\beta }\langle +|$$

Therefore

(10) $$\begin{array}{l} \langle \phi |\psi \rangle =-\frac{i}{2}\left(\cos\theta e^{-i\left(\alpha +\beta \right)}+i\sin\theta e^{i\left(\alpha -\beta \right)}\right)+\frac{i}{2}\left(\cos\theta e^{i\left(\alpha +\beta \right)}+i\sin\theta e^{-i\left(\alpha -\beta \right)}\right)\\ \approx -\frac{i}{2}\left[\left(\cos\theta +\sin\theta \sin2\alpha \right)e^{-i\left(\alpha +\beta \right)}-\left(\cos\theta -\sin\theta \sin2\alpha \right)e^{i\left(\alpha +\beta \right)}\right] \end{array}$$

In this system, we choose to observe the optical rotation, so the observation operator is $\hat{A}=\left|+\rangle \langle +\left|-\right|-\rangle \langle -\right|$. We can use *α*, *β*, and *γ* to represent weak values *A_w_* as follows:

(11) $$A_{w}=\frac{\langle \phi \left|A\right|\psi \rangle }{\langle \phi |\psi \rangle }\approx \frac{1+\gamma e^{2i\left(\alpha +\beta \right)}}{1-\gamma e^{2i\left(\alpha +\beta \right)}}$$

where $\gamma =\frac{\cos\theta -\sin\theta \sin2\alpha }{\cos\theta +\sin\theta \sin2\alpha }$, the imaginary part of *A_w_* is

(12) Im(Aw)≈2γsin(2α+2β)1+γ2−2γcos(2α+2β)

We can get the center wavelength offset as [10]

(13) δλ=−2πk(△λ)2λ0Im(Aw)≈−2πk(△λ)2γsin(2α+2β)λ0(1+γ2−2γcos(2α+2β))

Therefore, we can amplify the value of *α* + *β* by measuring the central wavelength. So, we can get the difference value of the optical substance solution before and after the sample cell.

We can also amplify *α* − *β*. We adjust the slide set so that the phase difference between *H* and *V* polarized light becomes △=π+2θ,θ≪1, and the operator $\hat{Q}$ of wave plate group can be expressed as

(14) $${\hat{Q}}^{\prime }=ie^{-i\theta }|H\rangle \langle H|-ie^{i\theta }|V\rangle \langle V|=\sin\theta |-\rangle \langle -|+\sin\theta |+\rangle \langle +|+i\cos\theta |+\rangle \langle -|+i\cos\theta |-\rangle \langle +|$$

From Formulas (5) and (6) we can get the states of circular polarization and weak value

(15) $$|\psi \rangle =\frac{\sqrt{2}}{2}\left(\sin\theta e^{-i\alpha }+i\cos\theta e^{i\alpha }\right)|-\rangle +\frac{\sqrt{2}}{2}\left(\sin\theta e^{i\alpha }+i\cos\theta e^{-i\alpha }\right)|+\rangle$$

(16) $$\langle \varphi |=-\frac{\sqrt{2}i}{2}e^{-i\beta }\langle -|+\frac{\sqrt{2}i}{2}e^{i\beta }\langle +|$$

(17) $$A_{w}^{\prime }=\frac{\langle \phi \left|A\right|\psi \rangle }{\langle \phi |\psi \rangle }\approx \frac{1+\gamma^{\prime }e^{-2i\left(\alpha -\beta \right)}}{1-\gamma^{\prime }e^{-2i\left(\alpha -\beta \right)}}$$

where $\gamma^{\prime }=\frac{\cos\theta +\sin\theta \sin2\alpha }{\cos\theta -\sin\theta \sin2\alpha }$. The imaginary part of *A_w_* is

(18) Im(Aw)≈−2γsin(2α−2β)1+γ2−2γcos(2α−2β)

The shift of central wavelength is

(19) δλ=−2πk(△λ)2λ0Im(Aw)≈2πk(△λ)2γsin(2α−2β)λ0(1+γ2−2γcos(2α−2β))

We can amplify the results of *α* − *β* by measuring the center wavelength.

Through the above two amplification methods, we can obtain the amplification results of the added and subtracted values of the optical rotation of the materials in SC-A and SC-B.

## 3. Experiment

### 3.1. Determination of High Sensitivity Detection Area of OR in the System

We adjust the system to a bimodal region with high sensitivity to the weak measurement system in the frequency domain. By adjusting the polarizer, the shift of the center wavelength of the system is recorded with the change of OR. At the same time, we also predict the equation of the effect of OR change on the system’s center offset.

**Figure 2:**
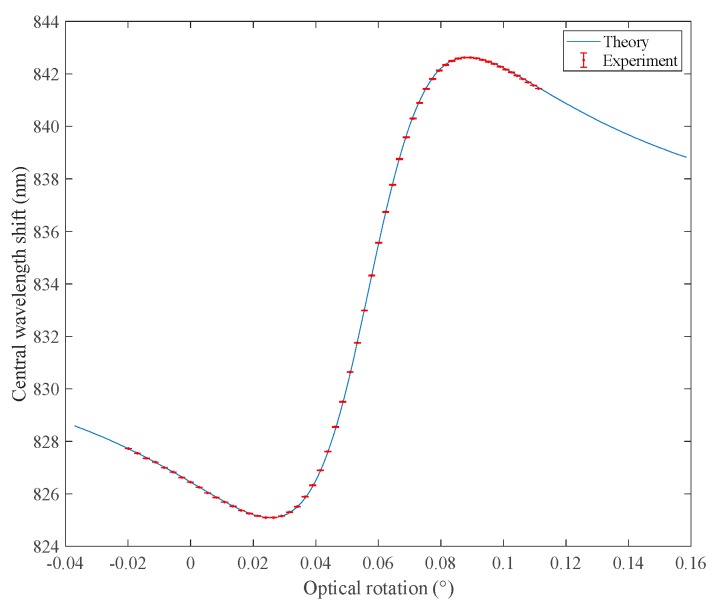
A simulation curve and experimental results of the central wavelength as a function of system optical rotation are obtained by adjusting the angle of the pre-selected polarizer.

From Figure 2, we can see that the center wavelength offset of the system decreases slowly with the increases of the OR change in the region near the bimodal region of weak measurement. When the center wavelength offset reaches the minimum value, it increases rapidly with the increase of the OR change. When the center wavelength offset of the system reaches the maximum value, it decreases slowly with the increases of the OR change. Therefore, the higher sensitivity of OR detection can be obtained in the sharp region, that is, the weak measurement bimodal region.

From Figure 2, we can see that the center wavelength offset of the system increases slowly with the increase of the OR change in the region near the bimodal region of weak measurement. When the center wavelength offset reaches the maximum value, it decreases rapidly with further increase of the OR change. When the center wavelength offset of the system reaches the minimum value, it increases slowly with the increase of the OR change. Therefore, the higher sensitivity of OR detection can be obtained in the sharp region, that is, the weak measurement bimodal region.

### 3.2. System OR Angle Detection Resolution

**Figure 3:**
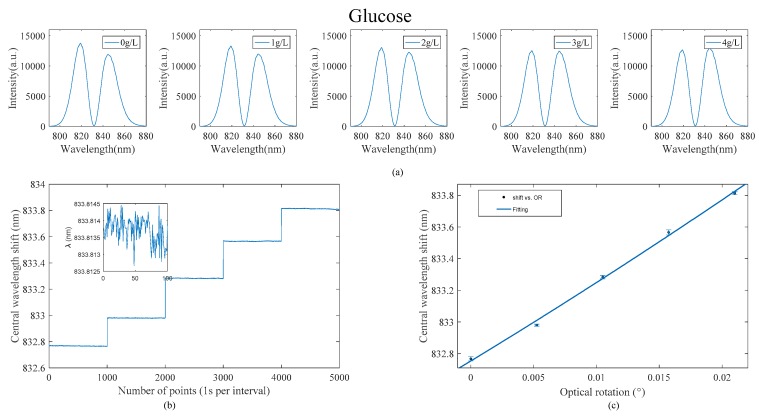
(**a**–**c**) The bimodal, central wavelength, and linear fit figures for different concentrations of glucose solution, respectively.

First, we set the system to the bimodal region and put the deionized water into cuvette B, and in cuvette A, the concentration of glucose solution is 0 g/L, 1 g/L, 2 g/L, 3 g/L, and 4 g/L, respectively. The experimentally detected two-peak spectra are shown in Figure 3a. We use the OCEAN VIEW program to record the data in the experiment. Because the bubbles generated during the process of replacing different concentrations of glucose solution will affect the optical path and cause the central wavelength to be irregularly offset, we only recorded data after each solution was replaced and the solution was stable. The relationship between the concentration of glucose solution and OR is +52.2, and sample cell length is 1 cm, so the rotation caused by the glucose solution of 4 g/L is 0.0209°.

As shown in Figure 3b,c, for this system, when the sample pool B is deionized water and the sample pool A is fed into the sample, the center wavelength of glucose solution changes to 1.05 nm when compared with deionized water. Because of the sensitivity formula of *δλ*/*λn*, the sensitivity of the system is obtained as 50.24 nm/degree. After the system is stable, 100 different data points are averaged, and the standard deviation is calculated. Figure 3b illustrates the changes in the 4 g/L glucose solution, and its standard deviation is 3.14 × 10^−4^ nm. By the formula $\sigma =3\sigma_{s}/(\delta \lambda /\delta n)$, the OR detection limit of the system can be calculated as 1.88 × 10^−50^.

### 3.3. Feasibility Analysis of the Sum and Difference of the Optical Rotation between the Sample and the Standard Product

#### 3.3.1. Response of the System to the Optical Activity of the Back Sample Cell at Different QWP Angles

**Figure 4:**
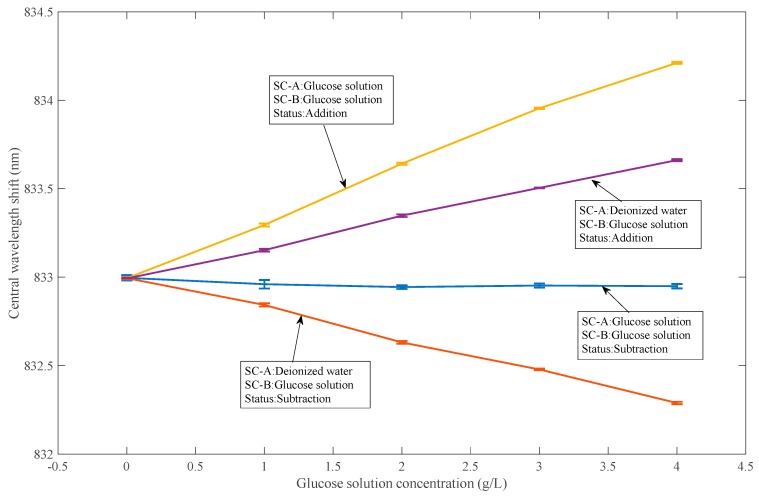
The feasibility of finding the sum and difference of the optical rotations of the solution in sample cell A and sample cell B.

The system’s response to the front sample cell has been experimentally tested in previous experiments. We only tested the optical response of the system to the back sample cell at different QWP angles. As shown in Figure 4, we added deionized water to sample cell A, and then adjusted the QWP to the position where the optical rotations produced by the solution in the pre- and post-sample cells are added. Glucose solutions with concentrations of 0 g/L, 1 g/L, 2 g/L, 3 g/L, and 4 g/L were added to sample cell B in turn. The shift of the center wavelength of the two peaks is recorded by spectrometer. It can be seen that the shift of the center wavelength varies with the concentration of the glucose solution as in the case where the deionized water SC-A is a glucose solution in SC-B.

As shown in Figure 4, we added deionized water to sample cell A, and then adjusted the QWP to the position where the optical rotations produced by the solution in the pre- and post-sample cells are subtracted. It can be seen that the shift in the center wavelength varies with the concentration of the glucose solution, as opposed to the case in SC-B, where the deionized water SC-A is a glucose solution.

#### 3.3.2. The Optical Rotation and Difference of Samples in the Sample Cell before and after the System are Obtained by Adjusting QWP

As shown in Figure 4, we first adjust the QWP to place the system in the position where the optical rotation of the sample in SC-A and SC-B is subtracted. Then, glucose solutions of concentrations 0 g/L, 1 g/L, 2 g/L, 3 g/L, and 4 g/L are sequentially added to the two sample cells, and the shift of the bimodal center wavelength is recorded by a spectrometer. As can be seen, when the system is in the state where the optical rotation of the front sample cell and the rear sample cell sample is subtracted, the change of the optical rotation of the samples cancel each other when they produce the same optical rotation. The center wavelength of the system does not change with changes in the glucose solution.

As shown in Figure 4, we first adjust the QWP so that the system is in the position where the optical rotation of the samples in SC-A and SC-B is added. Then, glucose solutions of concentrations 0 g/L, 1 g/L, 2 g/L, 3 g/L, and 4 g/L were sequentially added to the two sample cells, and the shift of the bimodal center wavelength was recorded by a spectrometer. As can be seen, when the system is in the state where the optical rotation of the front sample cell and the rear sample cell sample are subtracted, when the front and rear sample cells are introduced into the same optical rotation sample, the changes in the optical rotation of the samples are superimposed on each other. The center wavelength of the system is changed to be twice the amount of glucose solution in SC-A and deionized water in SC-B.

## 4. Determination of Enantiomer Content in Chiral Molecules

### 4.1. Ways to Detect the Ratio of Chiral Molecules to Enantiomeric Impurities

First, we placed deionized water in both sample cells and recorded the system’s bimodal center wavelength position A at this time. We placed a solution of T g/L of pure chiral molecules in sample cell B, and then placed the T g/L sample solution to be tested in sample cell A. Then, we changed the position of the difference in the optical rotation change caused by the QWP to the two sample cells of the system, and recorded the system bimodal central wavelength position at this time as M. Finally, we adjusted the position of the sum of the optical rotation changes caused by the QWP to the two sample cells of the system and recorded the center wavelength position N of the system at this time. The ratio of chiral molecules to their enantiomeric impurities in the sample to be tested is |(M − L)/(N − L)|.

### 4.2. Detecting the Ratio of Enantiomer Impurity of L-Proline in Chiral Molecule D-Valine

**Figure 5:**
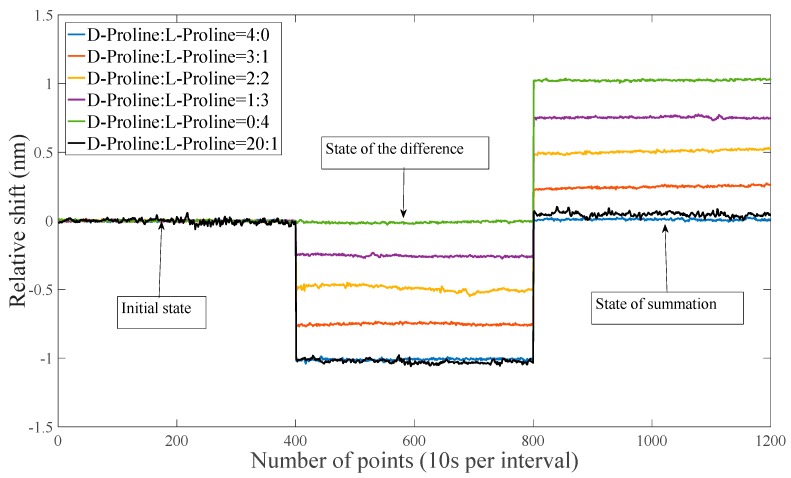
The figure shows the relative shifts in the central wavelengths of the different ratios of L-proline and D-proline in the two states of summation and difference.

Similarly, as shown in Figure 5, we first put deionized water in two sample cells and recorded the center wavelength of the system at this time. Here, we set the value to 0 for convenience. One g/L of D-valine solution was placed in the sample cell B, and then 1 g/L of the sample to be tested (D-valine containing L-proline impurity) was placed in sample cell A. Then, we modulate the position of the difference in the optical rotation change caused by the QWP to the two sample cells of the system and recorded the system’s bimodal central wavelength position at this time as M. Finally, we adjusted the position of the sum of the optical rotation changes caused by the QWP to the two sample cells of the system and recorded the center wavelength position N of the system at this time. The ratio of chiral molecules to their enantiomeric impurities in the sample to be tested is |M/N|.

As shown in the figure, we performed experiments in which the sample to be tested was 0:4, 1:3, 2:2, 3:1, and 4:0 of the L-proline impurity in D-valine. At these five impurity levels, the calculated results of the system center wavelength change caused by the subtraction and addition of the solution rotations in the two sample cells are shown in the table. As shown in Table 1, the ratio of the impurity content in the chiral molecule calculated by the central wavelength change is almost identical to the ratio in the sample that we set. It can be seen that the ratio of the enantiomeric magazines in the chiral molecules can be quickly detected by the method of the ratio of the sampled optical summation value to the difference between the two sample cells.

### 4.3. System Detection Limit

In order to determine the limits of system detection, as shown by the black line in Figure 5, we performed experiments in which the sample to be tested was 1:20 of the L-proline impurity in D-valine. Similarly, we set the value to 0 for convenience. 1.05 g/L of D-valine solution was placed in the sample cell B, and then 1.05 g/L of the sample to be tested (D-valine containing L-proline impurity) was placed in the sample cell A. By rotating the QWP we get the difference state, M, of −1.024 nm and the summation state, N, of 0.0477 nm. |M/N| is 1:21.47 with an error of 7.47%. So we were able to determine that the system could detect a mixture with an impurity content of 5%. That is, we believe that the detection limit of impurities is 5%. At the same time, the influence of noise in the system on the system is reduced by increasing the value of the sample solution and standard solution concentration T g/L at the time of detection. So, we can improve the detection accuracy of the system by increasing the value of T.

## 5. Discussion

Current frequency domain weak measurement technology is three orders of magnitude higher than the traditional interferometric measurement technology, so it has broad application prospects in biosensors. In our work, because the sensitivity of the system increases with the increase of the weak value, we obtain a larger weak value at the cost of losing a certain degree of light intensity. However, the loss of light intensity causes the influence of external noise on the system to become larger, which reduces the resolution of the system, so we can use a higher power source to improve the resolution of the system. At the same time, the sensitivity of the system is also affected by the length of the two sample cells and the sample solution and standard solution concentration T g/L, so we can also improve the detection accuracy of the system by increasing the length of the sample cell or concentration T g/L.

In this paper, the weak value enhancement method obtained better results than standard methods. This result is controversial according to some theoretical results, such as [25,26], which we think is due to noise [27]. In practical measurements, the noise model is relatively more complex and is in dispersed forms. Under the condition of this paper, the effect of weak value amplification is still effective and practical.

## 6. Conclusions

In this paper, we present a self-referential weak measurement system that can quickly detect the enantiomeric impurity content of chiral molecules. Because the OR can modify the angle of the selected state before and after the weak measurement system, the OR can be detected by the weak measurement system. In our work, we obtained the sum and difference of the OR of the material in the two sample cells in the system by adjusting the QWP angle. Thus, we can detect the proportion of enantiomeric impurities in the chiral molecule in the sample to be tested. The resolution of the optical rotation of the system was experimentally determined as 3.76 × 10^−50^ cm^2^. We verified the feasibility of our system by calculating the L-proline impurity content in D-valine. The self-based weak sensor has the advantages of being fast, simple, low cost, and having high stability and robustness. It is of great significance in the detection of drugs and food.

## Figures and Tables

**Table 1:** Comparison of settings and test results.

Setting Ratio (D-Proline:L-Proline)	Initial State (nm) (SC-A:Water SC-B:Water)	Difference State M (nm)	Summation State N (nm)	Test Results |M/N|
Pure D-proline	0	−1.0095	0.01	≈Pure D-proline
3:1	0	−0.7515	0.2468	≈3:1
2:2	0	−0.4919	0.5052	≈1:1
1:3	0	−0.255	0.7529	≈1:3
Pure L-proline	0	−0.0105	1.0242	≈Pure L-proline
